# GSNO as a Modulator of Vascular Tone in Human Saphenous Veins: Potential Implications for Graft Spasm

**DOI:** 10.3390/life15071139

**Published:** 2025-07-19

**Authors:** Deniz Kaleli Durman, Nurdan Dağtekin, Erkan Civelek, Taner İyigün, Önder Teskin, Birsel Sönmez Uydeş Doğan

**Affiliations:** 1 Department of Pharmacology, Faculty of Pharmacy, Istanbul University, Istanbul 34116, Türkiye; nurdandagtekin@hotmail.com (N.D.); erkan.civelek@istanbul.edu.tr (E.C.); sonmezdo@istanbul.edu.tr (B.S.U.D.); 2 Department of Cardiovascular Surgery, Mehmet Akif Ersoy Thoracic and Cardiovascular Surgery Training Research Hospital, Istanbul 34303, Türkiye; taneriyi@gmail.com; 3 Department of Cardiovascular Surgery, Faculty of Medicine, Biruni University, Istanbul 34015, Türkiye; oteskin@gmail.com

**Keywords:** S-nitrosoglutathione, human saphenous vein, vasorelaxation, nitric oxide, potassium channels

## Abstract

S-nitrosoglutathione (GSNO), a promising S-nitrosothiol, has been recognized for its ability to modulate vascular tone through its vasodilatory, antiplatelet, and antiproliferative effects. However, data on its vasodilatory effects in human vessels remain limited, and its mechanisms of action have yet to be fully elucidated. In this study, we aimed to investigate the vasorelaxant effect of GSNO and its underlying mechanisms, with particular focus on the soluble guanylate cyclase (sGC)/nitric oxide (NO) pathway and potassium channels in isolated human saphenous veins (SVs) obtained from patients undergoing coronary artery bypass grafting (CABG). GSNO (10^−8^–10^−4^ M) produced concentration-dependent relaxations in SV rings precontracted with phenylephrine. These relaxations were unaffected by NO synthase inhibition with L-NAME (10^−4^ M, 30 min) or NO scavenging with PTIO (10^−4^ M, 30 min), but were significantly reduced by the sGC inhibitor, ODQ (10^−5^ M, 30 min). Inhibition of ATP-sensitive (glibenclamid; 10^−5^ M, 30 min.), high-conductance Ca^2+^-activated (charybdotoxin; 10^−7^ M, 30 min), small-conductance Ca^2+^-activated (apamin; 10^−6^ M, 30 min), or voltage-dependent (4-aminopyridine; 10^−3^ M, 30 min) potassium channels did not alter the maximum relaxant responses to GSNO. Furthermore, pretreatment with GSNO (10^−4^ M, 30 min) significantly attenuated both the contractile response and sensitivity to phenylephrine. Collectively, these findings demonstrate that GSNO exerts acute vasorelaxant and modulatory effects in human SV primarily via cGMP-dependent mechanisms, highlighting its potential as a local therapeutic agent for preventing graft spasm in CABG.

## 1. Introduction

Given the critical regulatory role of nitric oxide (NO) in maintaining vascular homeostasis, considerable interest has been directed toward the development of exogenous NO-based therapeutic strategies for various cardiovascular diseases [1,2,3,4,5,6,7]. Among these, S-nitrosothiols have been recognized as novel NO donors for modulating vascular tone. These offer several advantages over classical NO donors, such as sodium nitroprusside (SNP) and organic nitrates, including a lower risk of systemic hypotension, development of vascular tolerance, and cytotoxicity, thereby presenting a more favorable option for therapeutic vascular modulation [8,9,10,11,12,13,14]. Based on numerous in vitro and in vivo studies as well as human investigations, S-nitrosoglutathione (GSNO) has gained attention as a promising therapeutic agent for cardiovascular therapy. Accordingly, GSNO has been shown to exert potent vasodilatory, antiplatelet, and antiproliferative effects via both cGMP-dependent and -independent mechanisms, thus highlighting its therapeutic potential in cardiovascular diseases [10,14,15,16,17,18,19,20,21,22,23].

Several animal studies have demonstrated that GSNO induces concentration-dependent vasorelaxant responses in various vascular beds including the isolated rat aorta [24,25,26,27,28], rat tail artery [29], rat mesenteric artery [30], rat femoral artery [30,31,32,33], and sheep mesenteric artery [34]. Similarly, a pronounced vasorelaxant effect of GSNO has been demonstrated in a limited number of studies conducted on human capacitance and resistance vessels under both in vivo and in vitro conditions [35,36]. These studies consistently reported that GSNO produces a sustained vasorelaxant response, particularly in vessels with endothelial dysfunction, and exhibits a comparable efficacy to that of classical nitrovasodilators [26,36,37].

In relation to the vasorelaxant mechanism of GSNO, experimental studies have demonstrated that activation of the soluble guanylate cyclase (sGC)/cGMP pathway constitutes the primary mechanism underlying its relaxant effects. Additionally, alternative mechanisms have also been proposed including the activation of various potassium channel subtypes and the S-nitrosylation of thiol groups on vascular smooth muscle proteins [8,9,38]. However, data regarding its vasodilatory effects in humans remain limited [35,36,39], and to date, no study has directly investigated the underlying mechanisms of GSNO-induced vasorelaxation in human vascular tissue.

The human saphenous vein (SV) is widely used as a bypass graft in coronary artery bypass grafting (CABG) due to its easy accessibility and favorable handling properties. However, SV grafts are known to exhibit poor endothelial function, high susceptibility to vasospasm during harvesting and in the early postoperative period, and generally display low long-term patency rates due to thrombosis and restenosis [40,41,42,43]. In this context, the local or perioperative administration of vasodilatory agents such as GSNO may improve graft performance by attenuating vasospasm and preserving endothelial integrity. Given its sustained and potent vasodilatory effects, along with its antiplatelet and antiproliferative properties, GSNO may serve as a promising local therapeutic agent for protecting vascular grafts, particularly in SVs with limited endogenous NO-releasing capacity.

This study aimed to investigate both the acute and preventive vasorelaxant effects of GSNO on isolated human SV rings and to elucidate its potential underlying mechanisms of relaxation, with a particular focus on the NO/sGC signaling pathway and potassium channel involvement. By addressing the current lack of mechanistic data in human SVs, this study provides valuable insights into the pharmacological actions of GSNO and supports its translational potential as a vascular therapeutic agent for graft spasm.

## 2. Materials and Methods

### 2.1. Harvesting and Preparation of Human Saphenous Veins

SV segments were obtained from patients undergoing coronary artery bypass operations. The use of discarded human vessels was approved by the Ethics Committee of the Istanbul University Institute of Cardiology (23 December 2015; 50.0.05.00/15). Informed consent was obtained from all patients who voluntarily participated in the study. The study was conducted in accordance with the principles outlined in the Declaration of Helsinki.

Caution was exercised during the harvesting of the vessels in order not to stretch and touch the endothelial surface, and no perioperative topical vasodilator agent was administered during the surgical procedure. After harvesting, the SV segments were placed into cold (4 °C) Krebs-Ringer bicarbonate solution and transferred to the laboratory immediately. The composition of the Krebs-Ringer bicarbonate solution was as follows (mM): NaCl 118.5, KCl 4.8, NaHCO_3_ 25, MgSO_4_·7H_2_O 1.2, CaCl_2_ 1.9, KH_2_PO_4_ 1.2, glucose 10.1, and disodium EDTA 0.026. After removing excess fat and adherent connective tissue, specimens were cut into rings 3–4 mm in length. Three to four rings were obtained from each SV specimen. SV rings were suspended between two stainless steel L-shaped hooks in 10 mL jacketed organ baths filled with Krebs-Ringer bicarbonate solution at 37 °C, aerated with 95% O_2_ and 5% CO_2_ (pH = 7.4). One hook was fixed at the bottom of the organ bath, while the other was connected via a micrometric manipulator to a force displacement transducer (Grass Model FT03; Grass Telefactor, West Warwick, RI, USA) for the measurement of isometric force.

### 2.2. Experimental Protocol

Each ring was initially stretched to an optimal resting tension of 2 g. Subsequently, rings were allowed to equilibrate for 2 h in bath solution, during which Krebs-Ringer solution was changed every 15 min. After the equilibration period, the viability of the SV rings was checked by potassium chloride (KCl, 40 mM), and rings that developed a tension of less than 2 g were discarded. Two consecutive KCl responses were obtained in each ring for standardization. Thereafter, the presence of functional endothelium was confirmed by the relaxation response to the endothelium-dependent vasodilator acetylcholine (ACh, 10^−4^ M) on phenylephrine (Phe, 3 × 10^−6^ M) precontracted human SV rings.

Although patients were receiving various cardiovascular medications pre/perioperatively, any residual pharmacological effects of such treatments are suggested to be minimal due to thorough washout periods after tissue harvesting and equilibration in the organ bath system.

#### 2.2.1. Investigation of the Relaxant Effect of GSNO on Isolated Human SV Rings

The effects of GSNO (10^−8^−10^−4^ M) were studied in a concentration-dependent manner on human SV rings precontracted submaximally with Phe (3 × 10^−6^ M). Additionally, the concentration-dependent vasorelaxant effect of SNP (10^−8^−10^−4^ M) was assessed to determine the maximum smooth muscle relaxation capacity of human SV rings. Time-matched control experiments were also performed to elucidate whether the precontractions elicited by Phe (3 × 10^−6^ M) were stable during the experimental period.

#### 2.2.2. Investigation of the Involvement of the NO Pathway in the Vasorelaxant Effect of GSNO

In order to evaluate the role of the NO pathway in the effects of GSNO, relaxant responses to GSNO (10^−8^−10^−4^ M) were studied in the presence and absence of the NO synthase inhibitor L-NAME (10^−4^ M, 30 min), the nitric oxide scavenger PTIO (10^−4^ M, 30 min), or the soluble guanylyl cyclase (sGC) inhibitor ODQ (10^−5^ M, 30 min) in human SV rings precontracted submaximally with Phe (3 × 10^−6^ M).

#### 2.2.3. Investigation of the Involvement of K^+^ Channels in the Vasorelaxant Effect of GSNO

For further evaluation of the mechanism of action, the possible involvement of potassium channels was investigated. Relaxant effects of GSNO (10^−8^−10^−4^ M) were studied in the absence (control) and presence of the selective inhibitors of ATP-sensitive K^+^ channels (glibenclamide; 10^−5^ M, 30 min), high conductance Ca^2+^-activated K^+^ channels (charybdotoxin; 10^−7^ M, 30 min), small conductance Ca^2+^-activated K^+^ channels (apamin; 10^−6^ M, 30 min), or non-selective voltage-dependent K^+^-channels (4-aminopyridine [4-AP]; 10^−3^ M, 30 min) in human SV rings precontracted submaximally with Phe (3 × 10^−6^ M).

The responses to GSNO were obtained in paired rings of the same vessel; one ring served as the control, while concentration–response curves to GSNO in the presence of the respective inhibitors were generated using the adjacent ring from the same vessel. The pharmacological inhibitors used to investigate the mechanisms of GSNO-induced vasorelaxation are listed in Supplementary Table S1.

#### 2.2.4. Investigation of the Preventive Effect of GSNO on Vascular Reactivity

The potential preventive effect of GSNO on vascular reactivity was evaluated by assessing the Phe-induced contractions in human SV rings. For this purpose, cumulative concentration–response curves to Phe (10^−8^ to 10^−4^ M) were obtained in the absence (control) or presence of GSNO (10^−4^ M, 30 min).

### 2.3. Statistical Analysis

All data are presented as the mean ± standard error of the mean (SEM). In all experiments, ‘n’ represents the number of patients from whom the vessels were obtained. Relaxation responses to GSNO, SNP, and ACh were expressed as percent (%) decreases of Phe-induced precontractions, while contractile responses to Phe were presented as percent (%) of KCl (40 mM)-induced contractions in that vessel ring. Emax denotes the maximal relaxation or the contraction responses to the relaxant and contractile agents. Sensitivities of the SV rings to relaxant and contractile agents were expressed as the effective concentration that elicited 50% of the maximum response (EC50 value) and were given as −logM (pEC50). pEC50 and Emax values were calculated for each concentration–response curve by probit regression analysis and presented as the mean ± SEM. Statistical analysis was performed using two-way repeated measures analysis of variance (ANOVA), followed by the Bonferroni post hoc test for comparison of the concentration–response curves and Student’s *t*-tests for the comparison of Emax and pEC50 values. *p* values less than 0.05 were considered statistically significant. GraphPad Prism software (version 9.4.0, Windows, Boston, MA, USA) was used for all statistical analyses.

### 2.4. Chemicals

ODQ and apamin were purchased from Tocris (Bristol, UK), and all other agents were obtained from Sigma-Aldrich Co. (St. Louis, MO, USA). GSNO was dissolved in distilled water, further diluted in Krebs-Ringer solution, and kept protected from light. ODQ, 4-aminopyridine, and glibenclamide were dissolved in dimethyl sulfoxide (DMSO) (final bath concentration < 0.1%). DMSO was determined to have no effect on the Phe-induced contractions. A stock solution of ACh was prepared in 0.001 N HCl, whereas SNP, Phe, and KCl were prepared in distilled water. All dilutions were freshly prepared in Krebs-Ringer solution on the day of each experiment.

## 3. Results

### 3.1. Patient Characteristics

The clinical characteristics of patients undergoing CABG and their medication regimens are presented in Table 1.

### 3.2. Relaxant Effects of GSNO on Isolated Human SV Rings

**Figure 1:**
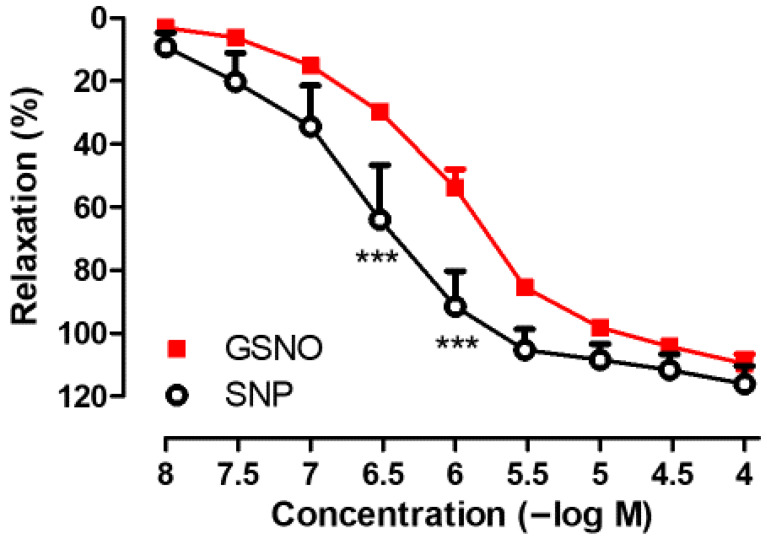
Concentration-dependent (10^−8^−10^−4^ M) vasorelaxant effects of GSNO and SNP on isolated human SV rings precontracted with Phe (3 × 10^−6^ M). *** *p* < 0.001; two-way repeated measures of ANOVA with Bonferroni post hoc test (n = 4–6, mean ± SEM).

As shown in Figure 1, GSNO (10^−8^−10^−4^ M) produced concentration-dependent relaxation responses in SV rings precontracted with Phe. The maximal relaxation response to GSNO was comparable to that induced by SNP (GSNO; Emax: 110.0 ± 3.06% vs. SNP; Emax: 115.2 ± 5.21%, *p* > 0.05, n = 4–6). However, the sensitivity to GSNO was significantly lower than that to SNP (GSNO; pEC50: 6.00 ± 0.01 vs. SNP; pEC50: 6.62 ± 0.28, * *p* < 0.05, n = 4–6).

In addition, the endothelium-dependent relaxation in response to Ach (10^−4^ M) was found to be 10.97 ± 2.39% (n = 6) in isolated human SV rings precontracted with Phe (3 × 10^−6^ M). Precontractions induced by Phe (3 × 10^−6^ M) were similar when assessing the relaxant effects of GSNO and SNP in SV rings. In time-matched control experiments, precontractions induced by Phe were found to be stable enough for the duration required to construct the concentration–response curves to GSNO or SNP.

### 3.3. Role of the NO Pathway in the Vasorelaxant Effect of GSNO

**Figure 2:**
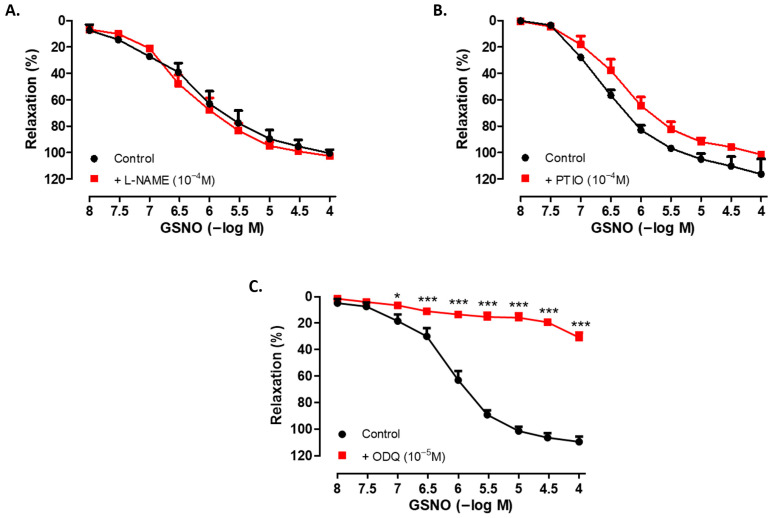
Concentration-dependent (10^−8^−10^−4^ M) vasorelaxant effect of GSNO in Phe-precontracted (3 × 10^−6^ M) isolated human SV rings in the absence (control) and in the presence of (**A**) L-NAME (10^−4^ M, 30 min), (**B**) PTIO (10^−4^ M, 30 min), and (**C**) ODQ (10^−5^ M, 30 min). * *p* < 0.05, *** *p* < 0.001; two-way repeated measures ANOVA with Bonferroni post hoc test (n = 4, mean ± SEM).

Incubation of SV rings with the NO synthase inhibitor L-NAME (10^−4^ M, 30 min) did not significantly alter the relaxant effect of GSNO (10^−8^−10^−4^ M) in isolated human SV rings precontracted with Phe (Figure 2A). Although a slight decrease was observed following pretreatment with the NO scavenger PTIO (10^−4^ M, 30 min), a significant difference was not observed in the relaxant responses to GSNO compared with the control group (Figure 2B). In contrast, pretreatment with the sGC inhibitor ODQ (10^−5^ M, 30 min) significantly decreased GSNO-induced relaxation in SV rings precontracted with Phe (Figure 2C). In addition, both the sensitivity to GSNO and the precontractions induced by Phe were found to be similar across all groups (Table 2).

### 3.4. Role of K^+^ Channels in the Vasorelaxant Effects of GSNO

**Figure 3:**
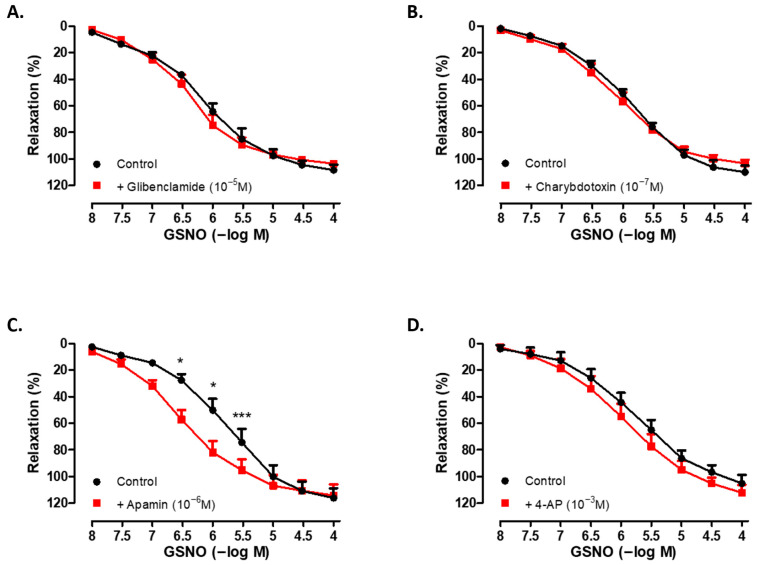
Concentration-dependent (10^−8^−10^−4^ M) vasorelaxant effect of GSNO on Phe-precontracted (3 × 10^−6^ M) isolated human SV rings in the absence (control) and presence of (**A**) glibenclamide (10^−5^ M, 30 min), (**B**) charybdotoxin (10^−7^ M, 30 min), (**C**) apamin (10^−6^ M, 30 min), and (**D**) 4-AP (10^−3^ M, 30 min). * *p* < 0.05, *** *p* < 0.001; two-way repeated measures ANOVA with Bonferroni post hoc test (n = 4–6, mean ± SEM).

As shown in Figure 3, incubation of human SV rings with selective inhibitors of ATP-sensitive K^+^ channels (glibenclamide; 10^−5^ M, 30 min) (Figure 3A), high-conductance Ca^2+^-activated K^+^ channels (charybdotoxin; 10^−7^ M, 30 min) (Figure 3B), small-conductance Ca^2+^-activated K^+^ channels (apamin; 10^−6^ M, 30 min) (Figure 3C), or non-selective voltage-dependent K^+^ channels (4-AP; 10^−3^ M, 30 min) (Figure 3D) did not significantly modify the maximum relaxation to GSNO compared with the corresponding control groups. Interestingly, the pEC50 values of GSNO were found to be higher in the presence of glibenclamide, charybdotoxin, or apamin compared with the respective controls. In addition, Phe-induced precontractions were found to be similar across all groups (Table 3).

### 3.5. Preventive Effect of GSNO on Vascular Reactivity

**Figure 4:**
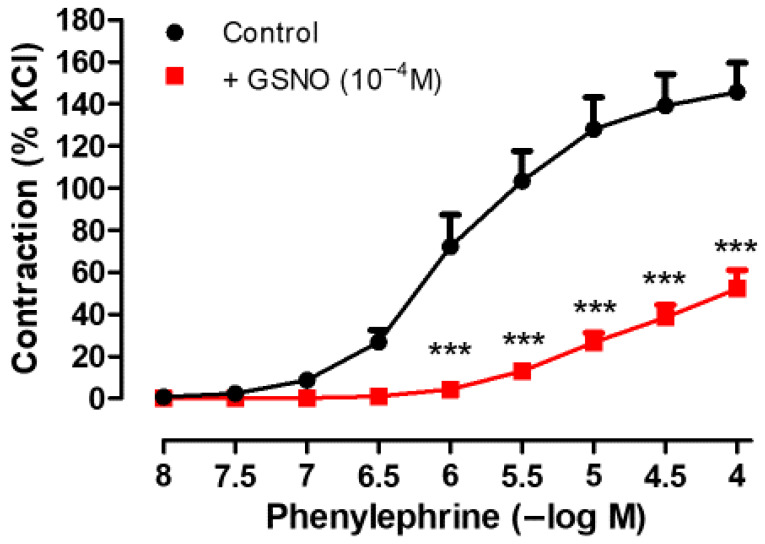
Effect of GSNO pretreatment (10^−4^ M, 30 min) on Phe-induced (10^−8^−10^−4^ M) contractile responses in isolated human SV rings. Contractile responses to Phe were expressed as percentages of the KCl (40 mM)-induced contractions in that vessel ring.*** *p* < 0.001 compared with the control, two-way repeated measures ANOVA with Bonferroni post hoc test (n = 4, mean ± SEM).

As shown in Figure 4, pretreatment of isolated human SV rings with GSNO (10^−4^ M, 30 min) significantly attenuated both the contractions and the sensitivity to Phe (10^−8^−10^−4^ M) compared with the control group (in the presence of GSNO; Emax: 61.54 ± 11.74% and pEC50: 4.92 ± 0.21 vs. control; Emax: 145.7 ± 14.08% and pEC50: 5.93 ± 0,12, * *p* ˂ 0.05, n = 4).

## 4. Discussion

The present study demonstrated that S-nitrosoglutathione (GSNO, 10^−8^−10^−4^ M) produced concentration-dependent relaxation in isolated human SV rings precontracted with Phe. The underlying mechanism of this vasorelaxant effect appears to be primarily mediated by the activation of sGC, while the involvement of endogenous NO synthesis and free NO release was negligible. Furthermore, potassium channels, including ATP-sensitive, high-conductance Ca^2+^-activated, small-conductance Ca^2+^-activated, and voltage-dependent K^+^ channels, did not appear to contribute significantly to this response. In addition, pretreatment of SV rings with GSNO significantly attenuated the contractile responses to Phe, indicating a modulatory effect of GSNO on vascular tone. Collectively, these findings indicate that GSNO is a potent vasorelaxant in human SV, acting predominantly through sGC/cGMP-dependent mechanisms. Given the susceptibility of human SV grafts to vasospasm, GSNO may represent a promising vasodilator for the prevention of SV graft spasm in CABG.

The vasorelaxant effects of S-nitrosothiols, including GSNO, have been demonstrated in numerous animal studies conducted on different vascular tissues such as the rat aorta [24,25,26,27,28], rat tail artery [29], rat mesenteric artery [30], rat femoral artery [30,31,32,33], and sheep mesenteric artery [34]. However, data regarding the vasorelaxant effects of GSNO on human vessels remain limited [35,36,39]. A comparative study evaluating human capacitance vessels (SV and dorsal hand vein) and resistance vessels (omental artery and forearm arterial bed) reported that GSNO was equipotent with the nitrovasodilator nitroglycerin (GTN) in relaxing human arterioles, but was less potent in human veins. Based on these in vitro and in vivo findings, an arterioselective vasodilator profile has been proposed for GSNO [35]. On the other hand, GSNO has been shown to produce a similar complete relaxation response, albeit with lower potency compared with GTN and SNP in isolated human SVs [35,36]. Consistent with these findings, the present study demonstrated that GSNO elicited immediate and complete vasorelaxation in Phe-precontracted human SV rings, exhibiting comparable efficacy but lower potency relative to SNP.

GSNO-induced relaxations were preserved in the presence of the NO synthase inhibitor L-NAME, in line with previous findings in human SV [36]. Considering the weak endothelial capacity as indicated by the low relaxation response to Ach in the SV rings, our results suggest that GSNO is an effective vasodilator in vessels with low basal NO capacity, comparable to other nitrovasodilators [36]. Supporting this, enhanced vasorelaxant responses to GSNO have been observed under conditions of impaired endothelial function or following NOS inhibition such as in isolated human IMA rings treated with L-NAME [36] and in renal lobar arteries obtained from hypertensive patients [39]. Overall, these findings suggest that GSNO may be particularly effective in vascular beds with endothelial dysfunction and could serve as a promising vasodilator for the management of SV graft spasm during the perioperative and postoperative periods of CABG.

The vasorelaxant effect of GSNO was not significantly altered in human SV rings in the presence of the NO scavenger PTIO, in line with a previous study conducted on isolated sheep mesenteric artery [34]. Earlier studies conducted in rat aortic rings reported no correlation between NO formation and the magnitude of relaxation induced by GSNO, suggesting that the spontaneous release of free NO does not account for the vasodilatory effect of S-nitrosothiols [24,25]. Supporting this, a previous study reported undetectable levels of NO release at GSNO concentrations ≤100 µM (pH 7.4) in rat aorta, which corresponds to the concentration range employed in the present study [25]. Therefore, in line with previous reports [10,29], we speculate that GSNO may directly transfer NO to reduced tissue thiols rather than through the release of free NO in human SVs, which requires further evaluation. Moreover, the sensitivity of S-nitrosothiols to reactive oxygen species (ROS) has been investigated in several studies. Some evidence suggests that antioxidants may enhance the vasorelaxant effects of S-nitrosothiols such as GSNO [8,24], whereas another study reported no such potentiation in the presence of ROS inhibitors [29]. Evaluating the effects of ROS inhibition in future studies could provide further mechanistic insights into GSNO-induced vasorelaxation in human SV rings.

The marked inhibition of GSNO-induced relaxation by the sGC inhibitor ODQ in human SV rings indicates that this response is primarily mediated via sGC activation. To the best of our knowledge, this is the first study to demonstrate the critical role of the sGC/cGMP pathway in mediating the vasorelaxant effects of GSNO in human SV. Supporting our findings, ODQ-sensitive relaxations to S-nitrosothiols have previously been reported in several vascular beds including the isolated rat aorta [27,44], rat femoral artery [33], and sheep mesenteric artery [34]. However, comparable evidence in human vessels has been lacking. Consistent with these prior studies, the inhibition of GSNO-induced relaxation by ODQ in human SV rings was incomplete. This observation suggests the potential involvement of sGC-independent mechanisms, such as potassium channel activation, in mediating the residual ODQ-insensitive component of the response [10]. Conversely, other reports have proposed that high concentrations of S-nitrosothiols, including GSNO, can overcome sGC inhibition by stimulating NO/cGMP generation, thereby excluding cGMP-independent mechanisms [25,44]. Notably, in the present study, 10 µM ODQ did not completely abolish the relaxation response to 100 µM GSNO in human SV. Indeed, GSNO at concentrations of 100 µM and above was shown to stimulate cGMP production in HEK-GC cell homogenates even in the presence of 10 µM ODQ, whereas cGMP generation was almost completely abolished with a higher ODQ concentration (100 µM). In contrast, 10 µM ODQ was found to be sufficient to fully inhibit GSNO (100 µM)-induced cGMP production in platelets [44]. These findings collectively suggest that the ability of GSNO to overcome ODQ-mediated inhibition may depend on multiple factors including the chemical properties of the S-nitrosothiol, its rate of NO release, the tissue or cell type studied, and the concentration of ODQ applied [10]. Although the present findings strongly suggest the involvement of sGC, further studies incorporating molecular assays, such as cGMP quantification or Western blot analysis of sGC expression, are warranted to confirm these results.

In a limited number of studies, S-nitrosothiols have been shown to regulate vascular tone not only through the classical NO–sGC/cGMP pathway, but also via the activation of potassium channels. In this context, the vasorelaxant effects of various S-nitrosothiols have been reported to involve the stimulation of intermediate- and small-conductance Ca^2+^-activated K^+^ channels in porcine microarteries [45]. Additionally, a previous study in rat aorta demonstrated a reduction in the sensitivity to GSNO without a change in the maximal relaxation following the blockade of potassium channels. It was therefore suggested that the vasorelaxant effect of GSNO is partially mediated through the activation of both K_ATP_ and low-conductance Ca^2+^-activated K^+^ channels [25]. However, to date, corresponding evidence in human vascular tissues remains lacking. Our findings demonstrated that the pretreatment of human SV rings with glibenclamide, charybdotoxin, apamin, or 4-AP did not significantly alter the maximal relaxation response to GSNO. Interestingly, an increased sensitivity to GSNO was observed in the presence of glibenclamide, charybdotoxin, and apamin; however, the underlying mechanism remains unclear. Our findings suggest that the activation of ATP-sensitive, high-conductance Ca^2+^-activated, small conductance Ca^2+^-activated, and voltage-dependent K^+^ channels is unlikely to play a significant role in mediating the vasorelaxant effect of GSNO in this vascular bed.

GSNO pretreatment has been shown to induce prolonged vasorelaxant responses in isolated rat aorta, rat mesenteric arteries, and porcine coronary arteries [26,37] as well as in human vascular tissues including the SV and IMA [36]. Consistent with these findings, the present study demonstrated that pretreatment with GSNO significantly attenuated both the maximal contractile response and the sensitivity to Phe in isolated human SV rings. A previous study proposed that the sustained vasorelaxant effects of S-nitrosothiols may be attributed to the S-nitrosation of cysteine residues, resulting in the formation of a reservoir of bioactive NO that is gradually released to maintain vascular relaxation over time [37]. Additionally, it has been suggested that S-nitrosothiols may be retained within subendothelial tissue compartments, where they undergo slow decomposition, continuously liberating NO, and thereby contributing to prolonged vasodilation [36]. The lack of inhibition by the NO scavenger PTIO suggests that free NO release is unlikely to contribute to the GSNO-induced prominent relaxations in human SV rings, at least within the concentration range used in this study. While the concept of an S-nitrosation-based NO reservoir remains plausible, its functional role in human veins requires further investigation using more selective NO scavengers and molecular assays. Notably, GSNO has been suggested to be particularly effective in vessels with endothelial dysfunction. In line with this, the marked inhibition of Phe-induced contraction observed in the present study, along with the previously reported long-lasting effect of GSNO (>180 min) following washout in human SV rings [36], supports its potential use of GSNO as a reliable vasodilator to counteract SV graft spasm during graft preparation or in the early postoperative period following CABG.

### Limitations of the Study

Despite providing valuable insights into the vasorelaxant effects of GSNO in human SV, this study had several limitations. The use of isolated human SV rings may not fully reflect the complex hemodynamic and cellular interactions that occur in vivo. In addition, the present study primarily focused on the acute and preventive effects of GSNO on vascular contractile responses; however, its long-term impact on SV reactivity remains to be elucidated. Furthermore, the sample size was relatively small, which may limit the generalizability of the findings. It should be noted that human SV segments were obtained from discarded tissue during CABG, which inherently limited the sample size and precluded the inclusion of additional pharmacological protocols. Although various pharmacological inhibitors were used to investigate the potential mechanisms of action, further studies are warranted to clarify the precise pathways involved in GSNO-induced vasorelaxation in human SVs. These may include molecular assays to directly confirm sGC involvement as well as investigations assessing the role of ROS inhibition. Another limitation is that subgroup analyses based on sex, age, and comorbidities such as hypertension could not be performed due to the limited sample size and the heterogeneous distribution of patients across these variables. This highlights the need for future studies designed to address these comparisons more robustly.

## 5. Conclusions

This study demonstrates that GSNO induces potent vasorelaxation in isolated human SV rings precontracted with Phe. The substantial relaxant responses remained unaltered in the presence of NO synthase inhibition, NO scavenging, or pharmacological blockade of various K^+^ channel subtypes, but were markedly attenuated by the sGC inhibitor ODQ. These findings suggest that GSNO-induced vasorelaxation in human SVs is primarily mediated through direct activation of the sGC/cGMP pathway. Furthermore, GSNO pretreatment significantly suppressed Phe-induced contractile responses, indicating a favorable modulatory effect on the contractile reactivity of the human SV.

Considering the potent vasodilatory effects demonstrated in this study, along with previous reports of GSNO’s sustained relaxant profile [26,36,37] as well as its antiplatelet [15,17] and antiproliferative effects on vascular smooth muscle cells [19], GSNO may be regarded as a promising therapeutic agent for managing graft spasm and improving long-term patency in SV grafts. However, its clinical utility needs to be confirmed through rigorous preclinical and early-phase clinical studies.

### Future Perspectives

The current experimental findings further support the development of innovative therapeutic strategies incorporating GSNO into implantable biomaterials such as polymer-coated stents, hydrogels, or nanoformulations [5,6,7,46,47]. Such localized delivery systems have the potential to enhance vasodilation, reduce thrombosis, inhibit neointimal hyperplasia, and prevent restenosis, thereby improving graft patency, particularly in SV grafts used for CABG. However, the clinical translation of these emerging approaches has yet to be established.

## Figures and Tables

**Table 1:** Clinical characteristics of the patients undergoing CABG.

Parameter	n (%)
Age (year)	60.43 ± 1.97
Number of patients Sex -Male -Female	29 21 (72.4%) 8 (27.6%)
Diseases	
Hypercholesterolemia	16 (55.2%)
Hypertension	22 (75.9%)
Diabetes mellitus	16 (55.2%)
Drug therapy	
β-blockers	26 (89.7%)
ACE Inhibitors	7 (24.1%)
Diuretics	19 (65.5%)
Drug therapy during operation	
Calcium channel blockers	1 (3.4%)
Nitrovasodilators	16 (55.2%)

n: number of patients, ACE: angiotensin-converting enzyme.

**Table 2:** Maximal relaxant effects (Emax) and pEC50 values of GSNO in the absence (control) or in the presence of L-NAME (10^−4^ M, 30 min), PTIO (10^−4^ M, 30 min), or ODQ (10^−5^ M, 30 min) in isolated human SV rings precontracted with Phe.

	Emax (%)	pEC50	Precontraction (g)
Control	99.34 ± 3.51	6.11 ± 0.24	6.89 ± 0.74
+L-NAME	101.50 ± 1.30	6.29 ± 0.20	7.63 ± 0.96
Control	112.20 ± 7.65	6.51 ± 0.16	4.98 ± 0.30
+PTIO	98.89 ± 1.51	6.28 ± 0.16	5.11 ± 0.78
Control	110.10 ± 3.18	6.10 ± 0.09	6.39 ± 0.78
+ODQ	33.32 ± 6.33 ***	5.27 ± 0.54	5.99 ± 0.73

All results are expressed as the mean ± SEM (n = 4); Emax values represent the percentage (%) of the maximum relaxation responses to GSNO on Phe-induced (3 × 10^−6^ M) precontractions. EC50 values are expressed as −log EC50 (pEC50). The Phe-induced precontraction levels in all groups are given as absolute contraction values (g). *** *p* < 0.001 compared with the corresponding control, paired Student’s *t*-test.

**Table 3:** The maximal relaxant effects (Emax) and pEC50 values of GSNO in the absence (control) or presence of glibenclamide (10^−5^ M, 30 min), charybdotoxin (10^−7^ M, 30 min), apamin (10^−6^ M, 30 min), or 4-AP (10^−3^ M, 30 min) in isolated human SV rings precontracted with Phe.

	Emax (%)	pEC50	Precontractions (g)
Control	108.80 ± 3.53	6.07 ± 0.18	6.44 ± 0.66
+Glibenclamide	103.00 ± 0.61	6.39 ± 0.15 **	6.22 ± 0.81
Control	110.30 ± 5.90	5.91 ± 0.10	4.47 ± 0.21
+Charybdotoxin	102.80 ± 2.85	6.07 ± 0.13 *	4.84 ± 0.72
Control	117.80 ± 7.23	5.80 ± 0.14	4.55 ± 0.23
+Apamin	112.50 ± 8.06	6.46 ± 0.17 *	4.69 ± 0.58
Control	104.70 ± 5.43	5.73 ± 0.14	5.32 ± 1.59
+4-AP	110.70 ± 5.43	5.99 ± 0.22	5.12 ± 0.45

All results are expressed as the mean ± SEM (n = 4–6). Emax values represent the percentage (%) of the maximum relaxation responses to GSNO on Phe-induced (3 × 10^−6^ M) precontractions. EC50 values are expressed as −log EC50 (pEC50), The Phe-induced precontraction levels in all groups are given as absolute contraction values (g). * *p* < 0.05, ** *p* < 0.01; compared with the corresponding controls, paired Student’s *t*-test.

## Data Availability

The data presented in this study are available on request from the corresponding author.

## Supplementary Materials

The following supporting information can be downloaded at: https://www.mdpi.com/article/10.3390/life15071139/s1, Table S1: List of pharmacological inhibitors, their targets, and concentrations used to investigate the mechanisms underlying GSNO-induced vasorelaxation in human saphenous vein rings.
