# Supplementary material for: GSNO as a Modulator of Vascular Tone in Human Saphenous Veins: Potential Implications for Graft Spasm
Source: Life (Basel). 2025 Jul 19;15(7):1139. doi: 10.3390/life15071139 (PMC12298686; doi:10.3390/life15071139)
Supplement: Supplementary file 1 [file life-15-01139-s001.zip › life-3730916-supplementary.pdf]

Supplementary Table S1: List of pharmacological inhibitors, their targets, and concentrations used to investigate the mechanisms underlying GSNO-induced vasorelaxation in human SV rings.

**Name**	**Chemical Description**	**Concentration**	**Action**
L-NAME	L-NG-Nitroarginine methyl ester	100 µM	nitric oxide synthase inhibitor
PTIO	2-Phenyl-4,4,5,5-tetramethylimidazoline-1-oxyl 3-oxide	100 µM	nitric oxide scavenger
ODQ	1H-[1,2,4]Oxadiazolo[4,3-a]quinoxalin-1-one	10 µM	soluble guanylyl cyclase inhibitor
4-AP	4-Aminopyridine	1 mM	voltage-dependent potassium channel inhibitor
Glibenclamide	5-Chloro-N-[4-(cyclohexylureidosulfonyl)phenethyl]-2-methoxybenzamide	10 µM	ATP-sensitive potassium channel inhibitor
Apamin	$C_{79}H_{131}N_{31}O_{24}S_4$	1 µM	small-conductance Ca^{+2}-activated K^+ channel inhibitor
Charybdotoxin	$C_{176}H_{277}N_{57}O_{55}S_7$	100 nM	high-conductance Ca^{+2}-activated K^+ channel inhibitor
